# Supplementary material for: Comparative Study on the Characterization of Myofibrillar Proteins from Tilapia, Golden Pompano and Skipjack Tuna
Source: Foods. 2022 Jun 10;11(12):1705. doi: 10.3390/foods11121705 (PMC9222683; doi:10.3390/foods11121705)
Supplement: Supplementary file 1 [file foods-11-01705-s001.zip › foods-1715325-supplementary.pdf]

Supplementary Information

Comparative study on the characterization of myofibrillar proteins from tilapia, golden pompano and skipjack tuna

Huibo Wang [a #], Zhisheng Pei [a, b #], Changfeng Xue [b], Jun Cao [a], Chuan Li [a, c*], Xuanri Shen[a, c*]

[a] Hainan Provincial Engineering Research Centre of Aquatic Resources Efficient Utilization in the South China Sea, School of Food Science and Engineering, Hainan University, Haikou 570228, China.

[b] School of Food Science and Engineering, Hainan Tropical Ocean University, Sanya 572022, China.

[c] Collaborative Innovation Center of Provincial and ministerial co-constructin for Marine Food Deep Processing, , Dalian Polytechnic University, Dalian 116034, China

# Huibo Wang and Zhisheng Pei contributed equally to this work.

*Corresponding author:

Chuan Li (E-mail: lichuan@hainanu.edu.cn, Tel. & Fax: +86-0898-66256495)

Xuanri Shen (E-mail: shenxuanri2009@163.com, Tel. & Fax: +86-0898-66196803)

Table S1 List of N-glycoproteins in TMP, GPMP and STMP resulting from a database search of LC-MS/MS data.

MP	Protein IDs	Protein names	Molecular weight [kDa]	Sequence length	Deamidation ^{18}O (N) site IDs[a]
TMP	A0A668RE17	Anaphylatoxin-like domain-containing protein	177.83	1573	0
	A0A669BS98	ATP-binding cassette, sub-family C (CFTR/MRP), member 12	104.97	939	14;15
	I3KGE9	BUB3 mitotic checkpoint protein	38.613	341	12;13
	A0A669ERR1	Carboxylic ester hydrolase	61.065	543	2
	A0A669F036	Collagen type VI alpha 3 chain	284.53	2595	19;20
	I3KXJ5	C-type lectin domain-containing protein	38.179	342	3
	Q9DE03	Decorin	39.807	359	6
	I3JFP9	Elastin microfibril interfacer 2b	107.72	969	25
	A0A669CDW4	Fibrillin 1	291.91	2682	11
	A0A668TBI7	Fibronectin	260.7	2345	7
	A0A668S9K0	Hemopexin	44.969	391	4
	A0A669ERK0	Hyaluronoglucosaminidase	142.74	1287	5
	I3KJ12	Late endosomal/lysosomal adaptor and MAPK and MTOR activator 1	17.161	160	27
	A0A668TMV8	Neuroguidin, EIF4E binding protein	39.839	350	9;10
	A0A669C429	Receptor protein-tyrosine kinase	99.831	892	17
	A0A669DZP5	Ribonuclease H	108.93	978	21
	A0A669EFF5	RNA helicase	69.068	614	1
	A0A669DV36	TGc domain-containing protein	48.112	427	18
	A0A669CY36	Uncharacterized protein	28.813	261	8
	A0A669B5G4	Uncharacterized protein	152.41	1392	16
	A0A669E7R7	Uncharacterized protein	101.67	910	22;23
	I3J8X4	Uncharacterized protein	20.661	193	24
	I3KEY4	Uncharacterized protein	531.61	4630	26

MP	Protein IDs	Protein names	Molecular weight [kDa]	Sequence length	Deamidation ^{18}O (N) site IDs[a]
GPMP	A0A3B4TZK6	1-phosphatidylinositol 4,5-bisphosphate phosphodiesterase	128.97	1129	49
	A0A3B4T4B1	Actin, alpha skeletal muscle A	41.595	374	2
	A0A3B4TWW5	ADP-ribosyl cyclase/cyclic ADP-ribose hydrolase	31.139	276	42
	A0A3B4XH64	AIG1-type G domain-containing protein	32.199	283	100;101
	A0A3B4YC69	Aminopeptidase	110.11	965	45
	A0A3B4UEE1	Apolipoprotein D	21.195	186	67
	A0A3B4UA97	ARID domain-containing protein	210.18	1958	61
	A0A3B4VQN0	Atriopeptidase	72.766	632	102;103
	A0A3B4WG85	Biglycan	42.238	372	78
	A0A3B4Z308	Calcium voltage-gated channel auxiliary subunit gamma 6	27.336	249	52
	A0A3B4XCM5	Carboxylic ester hydrolase	65.742	594	60
	A0A3B4U3M0	Cartilage associated protein	46.557	396	51
	A0A3B4XNT1	Cathepsin L1-like	36.788	330	120;121
	A0A3B4WEV7	CD276 molecule	32.199	298	24
	A0A3B4VBG9	Choline transporter-like protein	80.054	707	91
	A0A3B4YJD7	Collagen alpha-1(XII) chain-like	205.15	1905	63;64;65
	A0A3B4XJB4	Contactin-1a-like	105.85	956	98
	A0A3B4Z9U7	C-type lectin domain-containing protein	28.277	249	71
	A0A3B4Y620	Decorin	39.92	361	10
	A0A3B4YH71	Fetuin-B-like	38.887	350	94
	A0A3B4UV63	Fibrillin 1	305.02	2814	79;80;81
	A0A3B4TUC9	Fibrinogen like 2	46.56	408	41
	A0A3B4VNN2	Fructose-bisphosphate aldolase	39.669	363	99
	A0A3B4TET2	G_PROTEIN_RECEP_F1_2 domain-containing protein	28.002	251	17;18
	A0A3B4UI57	GPI ethanolamine phosphate transferase 2	107.11	966	69
	A0A3B4X4Y3	G-protein coupled receptor family C group 6 member A-like	105.69	942	110;111;112
	A0A3B4TSB4	Growth differentiation factor 10	50.749	450	37

MP	Protein IDs	Protein names	Molecular weight [kDa]	Sequence length	Deamidation ^{18}O (N) site IDs[a]
GPMP	A0A3B4X2X3	Hemopexin	47.346	412	108
	A0A3B4X514	Hepatocyte growth factor-like protein	80.969	707	0
	A0A3B4X3U3	HLA class II histocompatibility antigen, DP alpha 1 chain-like	26.456	239	109
	A0A3B4YJR3	Ig-like domain-containing protein	39.697	346	126
	A0A3B4WK93	Integrin beta	88.595	799	46;47;48
	A0A3B4WPS9	Integrin_alpha2 domain-containing protein	121.55	1101	106
	A0A3B4WUR7	Junctional adhesion molecule C-like	33.819	302	92
	A0A3B4TLR4	Laminin subunit alpha 2	346.2	3168	25;26;27
	A0A3B4U5E2	Laminin subunit alpha 4	203.37	1841	53;54;55;56;57;58
	A0A3B4TU85	Laminin subunit alpha 5	392.19	3573	39;40
	A0A3B4XJF1	Laminin subunit gamma 1	160.13	1454	113;114;115;116;117;118;119
	A0A3B4TJS0	Leucine-rich repeats and IQ motif containing 1	190.6	1647	22
	A0A3B4T904	Lumican	38.246	342	6;7;8;9
	A0A3B4TNT9	Lysosomal associated membrane protein 2	45.122	418	31
	A0A3B4Y6Y9	Lysosome-associated membrane glycoprotein 1-like	27.027	252	36
	A0A3B4WBG3	Matrilin 3	36.808	333	5
	A0A3B4WKN3	Matrix metalloproteinase-14-like	63.376	562	34
	A0A3B4V753	Mimecan	31.553	280	89
	Q9IBD7	Myosin heavy chain	221.76	1938	75;82;83;84;85
	A0A3B4YPF2	Myosin heavy chain, fast skeletal muscle	221.92	1935	73;74;75
	A0A3B4UWX2	Myosin heavy chain, fast skeletal muscle-like	221.83	1939	75;82;83;84;85
	Q9IB25	Myosin light chain 2	19.034	170	128
	A0A3B4VK05	Myosin light chain 3, skeletal muscle isoform	16.512	149	96
	A0A3B4VEB2	Myosin motor domain-containing protein	221.02	1928	75;82
	A0A3B4VQR6	Myosin-1B-like	225.08	1953	75;104
	A0A3B4XDW3	Myosin-7-like	223.08	1931	82
	A0A3B4TFV0	Neuronal pentraxin-1-like	47.205	423	19;20

MP	Protein IDs	Protein names	Molecular weight [kDa]	Sequence length	Deamidation ^{18}O (N) site IDs[a]
GPMP	A0A3B4UZV8	Peptidylprolyl isomerase	51.468	455	86
	A0A3B4UEM5	Phosphate regulating endopeptidase homolog X-linked	85.15	745	68
	A0A3B4XE03	Probable acyl-CoA dehydrogenase 6	47.865	430	35
	A0A3B4YTB2	Prostate stem cell antigen-like	13.748	130	90
	A0A3B4UJW1	Protein GPR108-like	64.289	566	70
	A0A3B4TP57	Protein phosphatase 1 regulatory subunit 37	129.55	1195	32
	A0A3B4YHV5	Sad1 and UNC84 domain containing 1	110.35	1001	59
	A0A3B4TQY7	Septin	49.479	426	33
	A0A3B4WRI7	SERPIN domain-containing protein	43.275	391	38
	A0A3B4U344	Shootin-1	31.932	274	50
	A0A3B4XCK8	Si:ch1073-459j12.1	76.213	672	95
	A0A3B4Y815	Sodium/potassium-transporting ATPase subunit beta	34.552	301	97
	A0A3B4X854	SpaA domain-containing protein	128.65	1173	93
	A0A3B4XVV0	Spectrin_like domain-containing protein	249.32	2191	122;123
	A0A3B4XGA4	Spindle assembly abnormal protein 6 homolog	67.637	590	105
	A0A3B4T2I9	Synapsin-2-like	51.336	468	1
	A0A3B4YKC3	Thrombospondin-1-like	129.93	1169	23
	A0A3B4XKH5	Transmembrane protein 245	92.055	831	66
	A0A3B4TIX1	Tumor necrosis factor receptor superfamily member 5-like	40.38	364	21
	A0A3B4TMG3	Uncharacterized protein	84.965	793	30
	A0A3B4YC93	Uncharacterized protein	224.24	1953	124;125
	A0A3B4XMW9	Uncharacterized protein	100.4	888	28;29
	A0A3B4T5T5	Uncharacterized protein	117.84	1056	3;4
	A0A3B4TXP8	Uncharacterized protein	162.22	1470	43;44
	A0A3B4WJQ0	Uncharacterized protein	210.03	1836	74;75;76;77;82;83;85
	A0A3B4WBE6	Uncharacterized protein	215.4	1883	75;82;83;84;85
	A0A3B4V1M8	Uncharacterized protein	46.918	417	87;88

MP	Protein IDs	Protein names	Molecular weight [kDa]	Sequence length	Deamidation ^{18}O (N) site IDs[a]
GPMP	A0A3B4XSU9	Vitamin K-dependent protein Z-like	50.069	453	11
	A0A3B4TE09	Voltage-dependent calcium channel gamma-6 subunit-like	24.317	222	15
	A0A3B4Z7D0	Voltage-dependent calcium channel subunit alpha-2/delta-1-like	120.94	1068	12;13;14
	A0A3B4TE24	Zinc finger and BTB domain-containing protein 5-like	44.148	408	16
STMP	A0A3B4B6P0	Alpha-1,4 glucan phosphorylase	97.016	840	29
	A0A3B4BHU1	BCL9 domain-containing protein	144.23	1344	32;33;34
	A0A3B3Z6N4	Carboxylic ester hydrolase	68.24	600	1;2
	A0A3B3ZI73	Decorin	27.695	249	9
	A0A3B4AKE6	Elongation factor 1-alpha	50.463	462	0
	A0A3B4ANI3	Integrin beta	81.572	727	23
	A0A3B4B8A5	Laminin_I domain-containing protein	13.127	116	30
	A0A3B4AP94	MRG domain-containing protein	54.412	474	24
	A0A3B4AG11	MYND-type domain-containing protein	41.421	368	18
	A0A3B4AY01	P66_CC domain-containing protein	44.215	412	25;26
	A0A3B4ALT9	Sema domain-containing protein	212.03	1891	22
	A0A3B3ZHL3	Uncharacterized protein	84.961	739	8
	A0A3B3ZI27	Uncharacterized protein	129.69	1167	11
	A0A3B4AB57	Uncharacterized protein	297.92	2738	15
	A0A3B4A8B5	Uncharacterized protein	145.2	1272	12;13;14
	A0A3B4AD31	Uncharacterized protein	51.035	444	16;17
	A0A3B4AKI7	Uncharacterized protein	190.6	1693	19;20;21
	A0A3B3ZCY4	Uncharacterized protein	41.944	377	3;4;5
	A0A3B4AZT3	Uncharacterized protein	42.032	377	27
	A0A3B4BD62	Uncharacterized protein	218.58	1987	31
	A0A3B3ZDS2	Uncharacterized protein	44.113	397	6;7
	A0A3B3ZI21	VWFA domain-containing protein	98.968	870	10

Table S2 List of N-glycopeptides in TMP, GPMP and STMP.

MP	Sequence	Length	Mass	Leading protein IDs	Score[b]	Deamidation ^{18}O (N) site IDs
TMP	PCHKLQDTFNR	11	1414.678	A0A668RE17	59.8	0
	SMPNCTSAFR	10	1169.496	A0A668S9K0	99.139	4
	EMNVAPDTTR	10	1132.518	A0A668TBI7	71.379	7
	MPAWNCLPHGGAINHREER	19	2244.043	A0A668TMV8	59.198	9;10
	DNVFTNSSGSR	11	1182.527	A0A669B5G4	137.95	16
	HMKNLENISR	10	1240.635	A0A669BS98	43.246	14;15
	AVAANGSSSGTQR	13	1204.58	A0A669C429	83	17
	QPCGNGTCK	9	1020.412	A0A669CDW4	122.19	11
	ANCTVLMTIK	10	1149.589	A0A669CY36	43.282	8
	VGQNISTK	8	845.4607	A0A669DV36	60.788	18
	EAAPEENGV	9	914.3981	A0A669DZP5	41.227	21
	EENATNQAEK	10	1132.5	A0A669E7R7	40.268	22;23
	ETENRPQK	8	1000.494	A0A669EFF5	40.103	1
	TTNATAADPR	10	1016.489	A0A669ERK0	56.729	5
	EEVNMTK	7	849.3902	A0A669ERR1	114.72	2
	AVQANNTALAQVIVLPNANSPQYNSVIQK	29	3064.631	A0A669F036	110.7	19
	EVVNTSQVDTPTPGLPPSK	19	1965.006	A0A669F036	190.86	20
	MTEAEIDALMAGQEDENGCVNYEAFVK	27	3033.304	I3J8X4	136.01	24
	LNTTEK	6	704.3705	I3JFP9	84.359	25
	DRNASNEK	8	932.4312	I3KEY4	45.718	26
	NPWLYNLNMKHWGMTGSNEYK	21	2582.184	I3KGE9	40.475	12;13
	SEDGNKQER	9	1061.474	I3KJ12	54.549	27
	IECANATQK	9	1033.486	I3KXJ5	90.657	3
	IADTNITEVPK	11	1199.64	Q9DE03	102.06	6
GPMP	GEIAGEPTKNGAGR	14	1355.679	A0A3B4T2I9	68.847	1
	DIKEKLCYVALDFENEMATAASSSSLEK	28	3148.494	A0A3B4T4B1	44.264	2
	LCYVALDFENEMATAASSSSLEK	23	2535.151	A0A3B4T4B1	244.57	2

MP	Sequence	Length	Mass	Leading protein IDs	Score[b]	Deamidation ^{18}O (N) site IDs
GPMP	RNTASTQNRDPK	12	1386.696	A0A3B4T5T5	59.35	3;4
	AGVFDNVTAELR	12	1290.657	A0A3B4T904	179.53	6
	LDANNVSHSNMPPDASNCLR	20	2210.98	A0A3B4T904	72.891	7;8;9
	ESCGPAELPGESNCTYFK	18	2044.851	A0A3B4TE09	107.53	15
	TFMNLTDCKK	10	1256.589	A0A3B4TE24	41.029	16
	STLSPTKKNNFR	12	1391.752	A0A3B4TET2	110.41	17;18
	LSTTEIYNLATCNSK	15	1713.824	A0A3B4TFV0	86.898	19;20
	TPEENLDELSAEGSMDPNR	19	2102.906	A0A3B4TIX1	41.401	21
	TDANDLYEIIMR	12	1452.692	A0A3B4TJS0	51.066	22
	AVAADANATAIDVLER	16	1598.826	A0A3B4TLR4	96.163	26
	NDKENLR	7	887.4461	A0A3B4TMG3	44.611	30
	YQEMNLEPNGTK	12	1422.645	A0A3B4TNT9	85.355	31
	RNFILAK	7	860.5232	A0A3B4TP57	72.618	32
	ELEEETNAFNRR	12	1506.706	A0A3B4TQY7	45.137	33
	VFAHESANRDMVSVNMFK	18	2080.982	A0A3B4TSB4	48.513	37
	AIENYNSTLDESR	13	1510.69	A0A3B4TU85	101.39	39
	DALNEAVNSTAR	12	1259.611	A0A3B4TU85	99.568	40
	VENITGVVNK	10	1071.592	A0A3B4TUC9	124.23	41
	NAVTNCTNASLK	12	1291.619	A0A3B4TWW5	93.096	42
	YNNFEVAEK	9	1112.514	A0A3B4TXP8	64.567	43;44
	KEMRANQAK	9	1074.56	A0A3B4TZK6	47.869	49
	ETVNSLLEEK	10	1160.593	A0A3B4U344	51.276	50
	DSEAFCNLNCSSVR	14	1657.683	A0A3B4U3M0	133.89	51
	KPVNNVTTNIMR	12	1385.745	A0A3B4U5E2	85.958	53
	VGVLNISTGAAANDR	15	1456.763	A0A3B4U5E2	132.62	54
	YVNDANITSITTLNLSQR	18	2022.038	A0A3B4U5E2	51.211	55;56;57
	YYNYTAHR	8	1086.488	A0A3B4U5E2	183.89	58
	QENKMEVEKK	10	1261.634	A0A3B4UA97	64.265	61

MP	Sequence	Length	Mass	Leading protein IDs	Score[b]	Deamidation ^{18}O (N) site IDs
GPMP	MLEAFNLTEK	10	1194.596	A0A3B4UBK5	113.71	62
	IEGTGVIEDMKNPAK	15	1600.813	A0A3B4UEE1	52.79	67
	EDYITNTSSAQAYR	14	1617.727	A0A3B4UEM5	155.53	68
	LSDLPAEPLSGSSPNSSR	18	1812.885	A0A3B4UI57	110.29	69
	LTLENETR	8	974.5033	A0A3B4UJW1	109.86	70
	ALQEAHQQVLDDLQAEEDKVNTLTK	25	2835.425	A0A3B4URR7	87.287	73
	CLAPKPQNTSK	11	1242.639	A0A3B4UV63	120.99	79
	NFYSDNGTCDGELTFNMTK	19	2212.904	A0A3B4UV63	82.34	80;81
	GQTVPQVNNSVMALCK	16	1744.86	A0A3B4UVX0	87.363	74
	ALQEAHQQTLDDLQAEEDKVNTLTK	25	2837.404	A0A3B4UWX2	479.45	82
	IAYLLGLNSADMLK	14	1520.827	A0A3B4UWX2	154.24	83
	LAQESIMDLENDKQQSDEK	19	2220.022	A0A3B4UWX2	416.07	84
	MEGDLNEMEIQLSHANR	17	1985.894	A0A3B4UWX2	217.09	75
	MQGSLEDQIVAANPLLEAYGNAK	23	2431.205	A0A3B4UWX2	284.25	85
	DDSMRSGMNGEMGIK	15	1626.68	A0A3B4UZV8	40.589	86
	QSLFGVLSEFNTHRNQCR	18	2192.055	A0A3B4V1M8	59.975	87;88
	SNDTYYLR	8	1030.472	A0A3B4V753	196.25	89
	VFNNSECEYSR	11	1403.578	A0A3B4VBG9	132.01	91
	MSEPEIDALMTGQEDENGSVHYEAFVK	27	3025.332	A0A3B4VK05	55.589	96
	GVVPLAGTNGETTTQGLDGLYER	23	2347.166	A0A3B4VNN2	103.88	99
	DIANATDTPEDR	12	1316.584	A0A3B4VQN0	147.74	102
	SCTNESLIELR	11	1320.634	A0A3B4VQN0	60.434	103
	LKEVTERLEDEEEVNAELTAK	21	2444.228	A0A3B4VQR6	56.569	104
	IERLENMVGR	10	1215.639	A0A3B4WBG3	48.561	5
	EGHDQLTDQAESFANR	16	1816.798	A0A3B4WEV7	123.28	24
	NIENGSLSYLPR	12	1361.694	A0A3B4WG85	107.83	78
	NKDPLNNSVVQLYQK	15	1758.926	A0A3B4WJQ0	151.26	76;77
	CHEGNGTFECGACK	14	1625.602	A0A3B4WK93	76.165	46

MP	Sequence	Length	Mass	Leading protein IDs	Score[b]	Deamidation ^{18}O (N) site IDs
GPMP	LINPCTGNQNCTSPFSYK	18	2099.941	A0A3B4WK93	117.93	47
	SAVGTLSSNSSNVIK	15	1462.763	A0A3B4WK93	158.56	48
	ATNEAIRK	8	901.4981	A0A3B4WKN3	47.849	34
	CQANQNGSQVECDLGNPVK	19	2116.927	A0A3B4WPS9	132.47	106
	GNTSMMIVLPDEGK	14	1490.711	A0A3B4WRI7	124.12	38
	EPATLLITNATR	12	1298.719	A0A3B4WUR7	123.75	92
	MEIDDLASNMEAVAK	15	1635.748	A0A3B4WV18	49.448	107
	GNTSHYIR	8	946.4621	A0A3B4X0P0	57.047	72
	SMPNCTSAFR	10	1169.496	A0A3B4X2X3	125.31	108
	NGENVTEAASLNVPYPNK	18	1915.928	A0A3B4X3U3	76.326	109
	VNFTFDNQNFYFDK	14	1797.8	A0A3B4X4Y3	61.65	110;111;112
	VNETTSGIPCQR	12	1360.641	A0A3B4X514	85.377	0
	LENMTAGTYTIR	12	1368.671	A0A3B4X854	94.616	93
	SVNEECPNITSIYSLGR	17	1937.915	A0A3B4XCK8	116.84	95
	NEVNMTK	7	834.3906	A0A3B4XCM5	116.54	60
	SLICLPMNLPGVHIAR	16	1789.97	A0A3B4XE03	42.001	35
	LTDDLDPYFLFNLSISEEDFQSLK	24	2848.37	A0A3B4XGA4	41.092	105
	GDNLNNQSVESYIEEECVDFVK	22	2587.138	A0A3B4XH64	50.088	100;101
	VDSSDTGNYSCIASSPSISK	20	2073.916	A0A3B4XJB4	130.98	98
	CDMCEENYFYNR	12	1699.607	A0A3B4XJF1	76.345	113
	FLGNQMLSYGQNLSLSFR	18	2074.031	A0A3B4XJF1	47.559	114;115
	IFANLTSLPPFDTK	14	1562.834	A0A3B4XJF1	108.37	116
	IPAINATIMAANEK	14	1455.776	A0A3B4XJF1	134.75	117
	KIPAINATIMAANEK	15	1583.871	A0A3B4XJF1	102.64	117
	VDIKPPGGTGEPNNMTLLAEEAR	23	2408.201	A0A3B4XJF1	159.81	118;119
	VNHTAVIEK	9	1009.556	A0A3B4XKH5	65.224	66
	LSENNRSVMK	10	1176.592	A0A3B4XMW9	61.999	28;29
	LEEPANFTNGTFK	13	1466.704	A0A3B4XNT1	87.308	120;121

MP	Sequence	Length	Mass	Leading protein IDs	Score[b]	Deamidation ^{18}O (N) site IDs
GPMP	SQMNVSHPLSNK	12	1340.651	A0A3B4XSU9	78.136	11
	SKALNENSTELQR	13	1488.753	A0A3B4XVV0	97.456	122;123
	IADTNITEIPK	11	1213.655	A0A3B4Y620	155.15	10
	TVQDVVNLQPNMTK	14	1585.813	A0A3B4Y6Y9	184.55	36
	PRPPTSNESIPEEAQPK	17	1875.933	A0A3B4Y815	82.543	97
	NATLASEASR	10	1018.504	A0A3B4YC69	108.57	45
	ELSERLEDEEEVNAELTAK	19	2203.049	A0A3B4YC93	92.039	124
	MEADLNEMEIQLSHANR	17	1999.909	A0A3B4YC93	79.82	125
	LANYFTLENITK	12	1425.75	A0A3B4YH71	80.763	94
	ALSPTGNITSAPR	13	1283.683	A0A3B4YHV5	75.416	59
	EANNITLDGYEVLGK	15	1634.815	A0A3B4YJD7	147.87	63
	GSESSYCFDGLTPDTLYNTTVYTQTPNLEGPGVSVK	36	3896.794	A0A3B4YJD7	63.008	64
	MLEAFNITDR	10	1208.586	A0A3B4YJD7	98.033	65
	AAELNQTLSR	10	1101.578	A0A3B4YJH2	169.12	25
	FENMTEELK	9	1139.517	A0A3B4YJH2	146.58	27
	QLNDSAAILDGILAEAK	17	1740.926	A0A3B4YJH2	194.85	127
	PIKTIMSSGLNSGIITQHK	19	2024.109	A0A3B4YJR3	51.841	126
	VVNSTTGPGEHLR	13	1365.7	A0A3B4YKC3	90.05	23
	EVNFPSSSSNTTVYTMTK	18	1991.915	A0A3B4YTB2	89.301	90
	TSCGPAELPGESNCTYFK	18	2016.856	A0A3B4Z308	120.9	52
	DDLNQTEK	8	961.4353	A0A3B4Z7D0	128.57	12
	DDLNQTEKK	9	1089.53	A0A3B4Z7D0	133.23	12
	FINTTQR	7	878.461	A0A3B4Z7D0	155.73	13
	GTVDCENCSR	10	1196.455	A0A3B4Z7D0	115.71	14
	WVDNTTLQHK	10	1240.62	A0A3B4Z9U7	43.168	71
	NMWSAFPPDVAGNVDYK	17	1909.867	Q9IB25	110.15	128

MP	Sequence	Length	Mass	Leading protein IDs	Score[b]	Deamidation ^{18}O (N) site IDs
STMP	AIPAADLSEQISTAGTEASGTGNMK	25	2419.154	A0A3B4B6P0	291.06	29
	AVAAKYNGELYNKR	14	1595.842	A0A3B3ZDS2	64.04	6;7
	DLVDSPLGSNNPLNMK	16	1712.84	A0A3B4BHU1	63.073	32;33;34
	DLYANNVLSGGTTMYPGIADR	21	2227.058	A0A3B3ZCY4	321.02	3;4
	ELGAINSTLSNQSK	14	1460.747	A0A3B4AD31	121.02	16;17
	FENMTEELK	9	1139.517	A0A3B4B8A5	110.41	30
	FNSTSIQCQK	10	1211.561	A0A3B4ALT9	201.39	22
	FRNSLKMLLTGGK	13	1463.828	A0A3B4BD62	54.066	31
	GTVDCENCSR	10	1196.455	A0A3B3ZI21	125.82	10
	IADTNITEIPK	11	1213.655	A0A3B3ZI73	110.38	9
	LCYVALDFENEMATAASSSSLEK	23	2535.151	A0A3B3ZCY4	308.03	5
	LEDDCFYINKRK	12	1599.772	A0A3B4AP94	103.56	24
	LKAESEDNQAELLK	14	1586.815	A0A3B3ZHL3	101.38	8
	LLNITDNIDEAER	13	1514.758	A0A3B3Z6N4	103.22	1
	LLNPCTGDQNCTSPFSYK	18	2100.925	A0A3B4B6F2	170.79	28
	LNYTLEEEK	9	1137.555	A0A3B3Z6N4	168.27	2
	MLEAFNITDR	10	1208.586	A0A3B4AB57	127.3	15
	NIKCNAVINEDPNNK	15	1741.842	A0A3B4AKI7	50.353	19;20;21
	QGKTQIVRSMYNEVAQTWNSHLTK	24	2818.418	A0A3B4AG11	48.823	18
	SAVGTLSSNSSNVIK	15	1462.763	A0A3B4ANI3	140.24	23
	VAPEEHPVLLTEAPLNPK	18	1953.057	A0A3B4AZT3	163.33	27
	VEQDIRRFFENLNPMGNR	18	2234.102	A0A3B4A8B5	57.885	12;13;14
	VETGVLKPGMVVTFAPVNVTTEVK	24	2514.377	A0A3B4AKE6	67.136	0
	VGNNASALVSSMK	13	1276.645	A0A3B4AY01	109.29	25;26
	VVNSTTGPGEHLR	13	1365.7	A0A3B3ZI27	85.958	11

a “Deamidation ^{18}O (N) site IDs”, conversion of Asn to (^{18}O) Asp resulting from ^{18}O/PNGase F treatment.

b “Score”, N-linked glycosylation localization score calculated using MaxQuant (version 1.6.1.0.); (Score for localization > 40 as the screening criteria for Number of Deamidation ^{18}O (N))

Table S3 List of N-glycosylation sites in TMP, GPMP and STMP.

MP	Protein IDs	Deamidation ^{18}O (N) Probabilities	Score for localization	Number of Deamidation ^{18}O (N)	Mass error [ppm]
TMP	A0A669F036	EVVN(1)TSQVDTPTPGLPPSK	190.86	1	0.88014
	A0A669B5G4	DNVFTN(1)SSGSR	137.95	1	-0.41215
	I3J8X4	MTEAEIDALMAGQEDEN(0.994)GCVN(0.006)YEAFVK	136.01	1	1.7315
	A0A669CDW4	QPCGN(1)GTCK	122.19	1	0.38481
	A0A669ERR1	EEVN(1)MTK	114.72	1	-0.4099
	A0A669F036	AVQAN(0.587)N(0.413)TALAQVIVLPNANSPQYNSVIQK	110.7	1	-2.3322
	Q9DE03	IADTN(1)ITEVPK	102.06	1	-0.50024
	A0A668S9K0	SMPN(1)CTSAFR	99.139	1	0.6074
	I3KXJ5	IECAN(1)ATQK	90.657		1.9827
	I3JFP9	LN(1)TTEK	84.359	1	-1.5374
	A0A669C429	AVAAN(1)GSSSGTQR	83	1	0.61228
	A0A669DV36	VGQN(1)ISTK	60.788	1	-0.51075
	A0A668RE17	PCHKLQDTFN(1)R	59.8	1	1.1019
	A0A668TMV8	MPAWN(1)CLPHGGAIN(1)HREER	59.198	2	1.5575
	A0A668TMV8	MPAWN(1)CLPHGGAIN(1)HREER	59.198	2	1.5575
	A0A669ERK0	TTN(1)ATAADPR	56.729	1	1.6749
	A0A668TBI7	EMN(1)VAPDTTR	54.776	1	-1.3473
	I3KJ12	SEDGN(1)KQER	54.549	1	-0.042516
	I3KEY4	DRN(0.119)ASN(0.881)EK	45.718	1	3.1277
	A0A669CY36	AN(1)CTVLMTIK	43.282	1	0.86085
	A0A669BS98	HMKN(1)LEN(1)ISR	43.246	2	0.80962
	A0A669BS98	HMKN(1)LEN(1)ISR	43.246	2	0.80962
	A0A669DZP5	EAAPEEN(1)GV	41.227		0.34635
	I3KGE9	N(0.373)PWLYN(0.315)LN(0.315)MKHWGMTGSN(0.998)EYK	40.475		-3.0698
	I3KGE9	N(0.373)PWLYN(0.315)LN(0.315)MKHWGMTGSN(0.998)EYK	40.475	2	-3.0698

MP	Protein IDs	Deamidation ^{18}O (N) Probabilities	Score for localization	Number of Deamidation ^{18}O (N)	Mass error [ppm]
TMP	A0A669E7R7	EEN(0.5)ATN(0.5)QAEK	40.268	1	1.4965
	A0A669E7R7	EEN(0.5)ATN(0.5)QAEK	40.268	1	1.4965
	A0A669EFF5	ETEN(1)RPQK	40.103	1	1.0384
GPMP	A0A3B4YJH2	QLN(1)DSAAILDGILAEAK	194.85	1	-0.97753
	A0A3B4Y6Y9	TVQDVVNLQPN(1)MTK	184.55	1	1.2903
	A0A3B4T904	AGVFDN(1)VTAELR	179.53	1	1.038
	A0A3B4YJH2	AAELN(1)QTLSR	169.12	1	0.73449
	A0A3B4WK93	SAVGTLSSN(0.991)SSN(0.009)VIK	158.56	1	0.7494
	A0A3B4UEM5	EDYITN(1)TSSAQAYR	155.53	1	-1.1357
	A0A3B4Y620	IADTN(1)ITEIPK	155.15	1	-0.54288
	A0A3B4YJD7	EAN(0.009)N(0.991)ITLDGYEVLGK	147.87	1	-0.46954
	A0A3B4VQN0	DIAN(1)ATDTPEDR	147.74	1	-0.99149
	A0A3B4XJF1	IPAIN(1)ATIMAANEK	134.75	1	0.56079
	A0A3B4U3M0	DSEAFCNLN(1)CSSVR	133.89		-0.23049
	A0A3B4Z7D0	DDLN(1)QTEKK	133.23	1	0.80701
	A0A3B4U5E2	VGVLN(1)ISTGAAANDR	132.62	1	0.68403
	A0A3B4WPS9	CQAN(0.001)QN(0.999)GSQVECDLGNPVK	132.47	1	0.94596
	A0A3B4VBG9	VFN(0.999)N(0.001)SECEYSR	132.01	1	0.18624
	A0A3B4XJB4	VDSSDTGN(1)YSCIASSPSISK	130.98	1	1.1056
	A0A3B4X2X3	SMPN(1)CTSAFR	125.31	1	-1.2595
	A0A3B4TUC9	VEN(1)ITGVVNK	124.23	1	-0.40301
	A0A3B4WRI7	GN(1)TSMMIVLPDEGK	124.12		1.2904
	A0A3B4WUR7	EPATLLITN(1)ATR	123.75	1	2.0073
	A0A3B4WEV7	EGHDQLTDQAESFAN(1)R	123.28	1	0.12558
	A0A3B4Z7D0	FIN(1)TTQR	122.79	1	0.018833
	A0A3B4UV63	CLAPKPQN(1)TSK	120.99	1	-0.97377

MP	Protein IDs	Deamidation ^{18}O (N) Probabilities	Score for localization	Number of Deamidation ^{18}O (N)	Mass error [ppm]
GPMP	A0A3B4Z308	TSCGPAELPGESN(1)CTYFK	120.9		1.8718
	A0A3B4WK93	LINPCTGN(0.03)QN(0.97)CTSPFSYK	117.93	1	-0.57935
	A0A3B4XCK8	SVNEECPN(1)ITSIYSLGR	116.84	1	3.5223
	A0A3B4XCM5	NEVN(1)MTK	116.54	1	-0.07523
	A0A3B4Z7D0	GTVDCEN(1)CSR	115.71	1	-0.63448
	A0A3B4V753	SN(1)DTYYLR	111.95	1	1.03
	A0A3B4TET2	STLSPTKKN(1)N(1)FR	110.41	2	-1.1011
	A0A3B4TET2	STLSPTKKN(1)N(1)FR	110.41	2	-1.1011
	A0A3B4UI57	LSDLPAEPLSGSSPN(1)SSR	110.29	1	0.77706
	A0A3B4UJW1	LTLEN(1)ETR	109.86	1	1.1182
	A0A3B4YC69	N(1)ATLASEASR	108.57	1	0.053649
	A0A3B4XJF1	IFAN(1)LTSLPPFDTK	108.37	1	0.26285
	A0A3B4WG85	NIEN(1)GSLSYLPR	107.83	1	0.96409
	A0A3B4TE09	ESCGPAELPGESN(1)CTYFK	107.53	1	1.0004
	A0A3B4VNN2	GVVPLAGTN(1)GETTTQGLDGLYER	103.88		3.1287
	A0A3B4TU85	AIEN(0.012)YN(0.988)STLDESR	101.39	1	-0.87512
	A0A3B4TU85	DALNEAVN(1)STAR	99.568	1	0.58484
	A0A3B4YJD7	MLEAFN(1)ITDR	98.033	1	-0.071978
	A0A3B4XJF1	VDIKPPGGTGEPN(0.028)N(0.972)MTLLAEEAR	97.974	1	0.57838
	A0A3B4XVV0	SKALN(0.342)EN(0.658)STELQR	97.456	1	0.54993
	A0A3B4XJF1	VDIKPPGGTGEPN(0.5)N(0.5)MTLLAEEAR	96.666	1	0.5057
	A0A3B4TLR4	AVAADAN(1)ATAIDVLER	96.163	1	0.84395
	A0A3B4X854	LEN(1)MTAGTYTIR	94.616	1	2.5195
	A0A3B4TWW5	NAVTN(0.995)CTN(0.005)ASLK	93.096	1	0.18355
	A0A3B4YC93	ELSERLEDEEEVN(1)AELTAK	92.039	1	-3.896
	A0A3B4YKC3	VVN(1)STTGPGEHLR	90.05	1	3.5494

MP	Protein IDs	Deamidation ^{18}O (N) Probabilities	Score for localization	Number of Deamidation ^{18}O (N)	Mass error [ppm]
GPMP	A0A3B4YTB2	EVNFPSSSSN(1)TTVYTMTK	89.301	1	-0.046664
	A0A3B4TFV0	LSTTEIYN(1)LATCN(1)SK	86.898		-3.3809
	A0A3B4TFV0	LSTTEIYN(1)LATCN(1)SK	86.898		-3.3809
	A0A3B4X514	VN(1)ETTSGIPCQR	85.377	1	4.2564
	A0A3B4TNT9	YQEMN(0.001)LEPN(0.999)GTK	85.355	1	-0.038255
	A0A3B4Y815	PRPPTSN(1)ESIPEEAQPK	82.543	1	1.055
	A0A3B4UV63	N(0.004)FYSDN(0.996)GTCDGELTFN(1)MTK	82.34	2	-1.1426
	A0A3B4UV63	N(0.004)FYSDN(0.996)GTCDGELTFN(1)MTK	82.34	2	-1.1426
	A0A3B4U5E2	KPVN(0.014)N(0.985)VTTN(0.001)IMR	81.799	1	0.47859
	A0A3B4YH71	LANYFTLEN(1)ITK	80.763	1	0.036037
	A0A3B4YC93	MEADLN(1)EMEIQLSHANR	79.82	1	-3.9804
	A0A3B4XNT1	LEEPAN(0.5)FTN(0.5)GTFK	78.934	1	-0.5087
	A0A3B4XNT1	LEEPAN(0.5)FTN(0.5)GTFK	78.934	1	-0.5087
	A0A3B4XSU9	SQMN(1)VSHPLSNK	78.136	1	-1.89
	A0A3B4UWX2	MEGDLN(1)EMEIQLSHANR	76.655		2.1863
	A0A3B4XJF1	CDMCEEN(0.002)YFYN(0.998)R	76.345	1	-0.56466
	A0A3B4X3U3	N(0.038)GEN(0.962)VTEAASLNVPYPNK	76.326	1	-0.099278
	A0A3B4YHV5	ALSPTGN(1)ITSAPR	75.416	1	0.17865
	A0A3B4YJH2	FEN(1)MTEELK	74.92	1	2.6462
	A0A3B4UBK5	MLEAFN(1)LTEK	73.985	1	-0.25426
	A0A3B4T904	LDAN(0.208)N(0.792)VSHSN(0.967)MPPDASN(0.033)CLR	72.891	2	4.4861
	A0A3B4T904	LDAN(0.208)N(0.792)VSHSN(0.967)MPPDASN(0.033)CLR	72.891	2	4.4861
	A0A3B4TP57	RN(1)FILAK	72.618	1	1.6872
	A0A3B4T904	LDAN(0.5)N(0.5)VSHSN(0.945)MPPDASN(0.054)CLR	69.352	2	3.7346
	A0A3B4T2I9	GEIAGEPTKN(1)GAGR	68.847	1	1.0716
	A0A3B4XKH5	VN(1)HTAVIEK	65.224	1	1.1536

MP	Protein IDs	Deamidation ^{18}O (N) Probabilities	Score for localization	Number of Deamidation ^{18}O (N)	Mass error [ppm]
GPMP	A0A3B4TXP8	YN(1)N(1)FEVAEK	64.567	2	-1.4896
	A0A3B4TXP8	YN(1)N(1)FEVAEK	64.567	2	-1.4896
	A0A3B4UA97	QEN(1)KMEVEKK	64.265	1	-1.7295
	A0A3B4YJD7	GSESSYCFDGLTPDTLYN(1)TTVYTQTPNLEGPGVSVK	63.008	1	0.50157
	A0A3B4XMW9	LSEN(0.5)N(0.5)RSVMK	61.999	1	2.9997
	A0A3B4XMW9	LSEN(0.5)N(0.5)RSVMK	61.999	1	2.9997
	A0A3B4U5E2	YYN(1)YTAHR	61.962	1	0.13967
	A0A3B4UWX2	IAYLLGLN(1)SADMLK	61.815	1	1.3627
	A0A3B4X4Y3	VN(0.333)FTFDN(0.333)QN(0.333)FYFDK	61.65		4.0979
	A0A3B4X4Y3	VN(0.333)FTFDN(0.333)QN(0.333)FYFDK	61.65		4.0979
	A0A3B4X4Y3	VN(0.333)FTFDN(0.333)QN(0.333)FYFDK	61.65		4.0979
	A0A3B4WK93	CHEGN(1)GTFECGACK	61.167	1	-0.34944
	A0A3B4VQN0	SCTN(1)ESLIELR	60.434	1	0.22536
	A0A3B4V1M8	QSLFGVLSEFN(1)THRN(1)QCR	59.975	2	-1.6165
	A0A3B4V1M8	QSLFGVLSEFN(1)THRN(1)QCR	59.975	2	-1.6165
	A0A3B4T5T5	RN(1)TASTQN(1)RDPK	59.35	2	-0.04599
	A0A3B4T5T5	RN(1)TASTQN(1)RDPK	59.35	2	-0.04599
	A0A3B4XVV0	SKALN(0.777)EN(0.223)STELQR	57.174	1	-0.88539
	A0A3B4X0P0	GN(1)TSHYIR	57.047	1	0.86541
	A0A3B4VQR6	LKEVTERLEDEEEVN(1)AELTAK	56.569	1	-3.2246
	A0A3B4UWX2	ALQEAHQQTLDDLQAEEDKVN(1)TLTK	56.131		-0.37211
	A0A3B4VK05	MSEPEIDALMTGQEDEN(1)GSVHYEAFVK	55.589	1	4.3791
	A0A3B4T4B1	LCYVALDFEN(1)EMATAASSSSLEK	52.909	1	2.5189
	A0A3B4UEE1	IEGTGVIEDMKN(1)PAK	52.79	1	-3.6341
	A0A3B4UWX2	LAQESIMDLEN(1)DKQQSDEK	52.298	1	0.33224
	A0A3B4UWX2	MQGSLEDQIVAAN(0.001)PLLEAYGN(0.999)AK	51.888		1.6706

MP	Protein IDs	Deamidation ^{18}O (N) Probabilities	Score for localization	Number of Deamidation ^{18}O (N)	Mass error [ppm]
GPMP	A0A3B4YJR3	PIKTIMSSGLN(1)SGIITQHK	51.841		4.3563
	A0A3B4U344	ETVN(1)SLLEEK	51.276	1	4.0744
	A0A3B4U5E2	YVN(1)DAN(1)ITSITTLN(1)LSQR	51.211		-3.5275
	A0A3B4U5E2	YVN(1)DAN(1)ITSITTLN(1)LSQR	51.211		-3.5275
	A0A3B4U5E2	YVN(1)DAN(1)ITSITTLN(1)LSQR	51.211		-3.5275
	A0A3B4TJS0	TDAN(1)DLYEIIMR	51.066	1	-0.033835
	A0A3B4XH64	GDN(0.157)LN(0.422)N(0.422)QSVESYIEEECVDFVK	50.088		1.2685
	A0A3B4XH64	GDN(0.157)LN(0.422)N(0.422)QSVESYIEEECVDFVK	50.088		1.2685
	A0A3B4WV18	MEIDDLASN(1)MEAVAK	49.448	1	-4.3488
	A0A3B4WBG3	IERLEN(1)MVGR	48.561	1	-1.7872
	A0A3B4TSB4	VFAHESANRDMVSVN(1)MFK	48.513		3.4649
	A0A3B4TZK6	KEMRAN(1)QAK	47.869	1	0.64051
	A0A3B4WKN3	ATN(1)EAIRK	47.849	1	-1.2292
	A0A3B4URR7	ALQEAHQQVLDDLQAEEDKVN(1)TLTK	47.745	1	-4.2433
	A0A3B4XJF1	FLGN(1)QMLSYGQN(1)LSLSFR	47.559	2	3.8341
	A0A3B4XJF1	FLGN(1)QMLSYGQN(1)LSLSFR	47.559	2	3.8341
	Q9IB25	N(1)MWSAFPPDVAGNVDYK	46.069		4.0441
	A0A3B4UVX0	GQTVPQVN(0.663)N(0.337)SVMALCK	45.915	1	-1.406
	A0A3B4TQY7	ELEEETN(0.893)AFN(0.107)RR	45.137	1	-3.961
	A0A3B4TMG3	NDKEN(1)LR	44.611	1	-2.2994
	A0A3B4WJQ0	N(0.054)KDPLN(0.473)N(0.473)SVVQLYQK	44.44		0.44922
	A0A3B4WJQ0	N(0.054)KDPLN(0.473)N(0.473)SVVQLYQK	44.44		0.44922
	A0A3B4Z9U7	WVDN(1)TTLQHK	43.168	1	0.94027
	A0A3B4XE03	SLICLPMN(1)LPGVHIAR	42.001	1	3.9478
	A0A3B4TIX1	TPEEN(1)LDELSAEGSMDPNR	41.401	1	-0.85896
	A0A3B4XGA4	LTDDLDPYFLFN(1)LSISEEDFQSLK	41.092	1	0.62611

MP	Protein IDs	Deamidation ^{18}O (N) Probabilities	Score for localization	Number of Deamidation ^{18}O (N)	Mass error [ppm]
GPMP	A0A3B4TE24	TFMN(1)LTDCKK	41.029	1	-4.1688
	A0A3B4UZV8	DDSMRSGMN(1)GEMGIK	40.589	1	2.2971
STMP	A0A3B4ALT9	FN(1)STSIQCQK	201.39	1	0.50615
	A0A3B4B6F2	LLNPCTGDQN(1)CTSPFSYK	170.79	1	-1.0188
	A0A3B3Z6N4	LN(1)YTLEEEK	168.27	1	-0.17486
	A0A3B4ANI3	SAVGTLSSN(0.962)SSN(0.038)VIK	140.24	1	0.015995
	A0A3B3ZI21	GTVDCEN(1)CSR	125.82	1	-0.94426
	A0A3B4AD31	ELGAIN(1)STLSN(1)QSK	121.02	2	1.2547
	A0A3B4AD31	ELGAIN(1)STLSN(1)QSK	121.02	2	1.2547
	A0A3B4B8A5	FEN(1)MTEELK	110.41	1	0.34599
	A0A3B3ZI73	IADTN(1)ITEIPK	110.38	1	-0.21372
	A0A3B4AY01	VGN(1)N(1)ASALVSSMK	109.29		0.19745
	A0A3B4AY01	VGN(1)N(1)ASALVSSMK	109.29		0.19745
	A0A3B4AP94	LEDDCFYIN(1)KRK	103.56	1	2.2035
	A0A3B3Z6N4	LLN(1)ITDNIDEAER	103.22	1	1.0141
	A0A3B3ZHL3	LKAESEDN(1)QAELLK	101.38	1	-2.2293
	A0A3B3ZI27	VVN(1)STTGPGEHLR	85.958	1	2.2848
	A0A3B4AB57	MLEAFN(1)ITDR	80.239	1	-0.28125
	A0A3B4AKE6	VETGVLKPGMVVTFAPVN(1)VTTEVK	67.136	1	-3.3203
	A0A3B3ZDS2	AVAAKYN(1)GELYN(1)KR	64.04	2	-1.3543
	A0A3B3ZDS2	AVAAKYN(1)GELYN(1)KR	64.04	2	-1.3543
	A0A3B4BHU1	DLVDSPLGSN(1)N(1)PLN(1)MK	63.073	3	-1.5945
	A0A3B4BHU1	DLVDSPLGSN(1)N(1)PLN(1)MK	63.073	3	-1.5945
	A0A3B4BHU1	DLVDSPLGSN(1)N(1)PLN(1)MK	63.073	3	-1.5945
	A0A3B4AZT3	VAPEEHPVLLTEAPLN(1)PK	58.674	1	-0.052307

MP	Protein IDs	Deamidation ^{18}O (N) Probabilities	Score for localization	Number of Deamidation ^{18}O (N)	Mass error [ppm]
STMP	A0A3B4B6P0	AIPAADLSEQISTAGTEASGTGN(1)MK	58.658	1	2.6068
	A0A3B4BD62	FRN(1)SLKMLLTGGK	54.066	1	-0.55383
	A0A3B3ZCY4	DLYAN(0.5)N(0.5)VLSGGTTMYPGIADR	53.491	1	2.5231
	A0A3B3ZCY4	DLYAN(0.5)N(0.5)VLSGGTTMYPGIADR	53.491	1	2.5231
	A0A3B3ZCY4	LCYVALDFEN(1)EMATAASSSSLEK	51.645	1	1.951
	A0A3B4AKI7	N(0.305)IKCN(0.305)AVIN(0.389)EDPN(1)N(1)K	50.353		-2.5027
	A0A3B4AKI7	N(0.305)IKCN(0.305)AVIN(0.389)EDPN(1)N(1)K	50.353	3	-2.5027
	A0A3B4AKI7	N(0.305)IKCN(0.305)AVIN(0.389)EDPN(1)N(1)K	50.353	3	-2.5027
	A0A3B4A8B5	VEQDIRRFFEN(1)LN(1)PMGN(1)R	49.081	3	1.0657
	A0A3B4A8B5	VEQDIRRFFEN(1)LN(1)PMGN(1)R	49.081	3	1.0657
	A0A3B4A8B5	VEQDIRRFFEN(1)LN(1)PMGN(1)R	49.081	3	1.0657
	A0A3B4AG11	QGKTQIVRSMYN(0.171)EVAQTWN(0.829)SHLTK	48.823	1	0.71099
